# From loops to caps: discriminating peptide binding to distinct G-quadruplex tetrads using 5-furyl-2′-deoxyuridine fluorescent probes

**DOI:** 10.1039/d5cb00247h

**Published:** 2025-11-10

**Authors:** Jack Barr, Tayler D. Prieto Otoya, Christine Cardin, Enrico Cadoni

**Affiliations:** a Organic and Biomimetic Chemistry Research Group, Ghent University, Krijgslaan 281 S4 B-9000 Ghent Belgium Enrico.Cadoni@UGent.be; b Department of Chemistry, University of Reading, Whiteknights Reading RG6 6AD UK

## Abstract

We report the use of 5-furyl-2′-deoxyuridine (5FU) as a fluorescent probe to distinguish ligand binding to distinct G-quadruplex tetrads. Site-specific incorporation of 5FU into T95-2T at the capping regions enabled discrimination of peptide-binding events *via* fluorescence turn-on, providing insights into tetrad preference and binding affinities using the 5FU-G4/RHAU peptide system.

## Abstract 2

Site-specific 5-furyl-2′-deoxyuridine (5FU) labels at G-quadruplex (G4) caps generate fluorescence turn-on readouts that resolve tetrad-specific binding of peptide RHAU23, enabling tetrad affinity determination on T95-2T and other parallel G4s.

## Graphical abstract

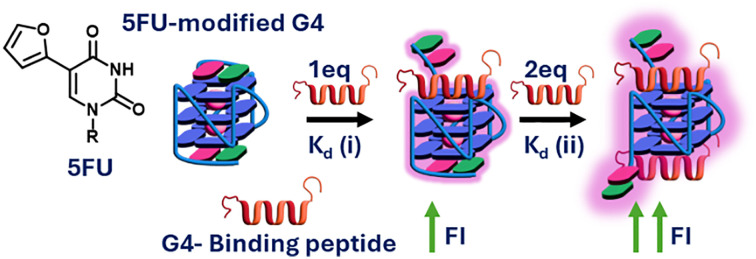

Guanine-rich nucleic acid sequences can fold into non-canonical secondary structures called G-quadruplexes (G4s).^1^ Over the past 25 years, G4s have been implicated in diverse biological processes, including transcriptional regulation, telomere maintenance, and RNA translation, making them attractive drug targets.^2^ Numerous ligands, particularly small aromatic molecules and, more recently, peptide-based ligands derived from G4-binding proteins (G4BPs), have been developed to modulate G4 activity.^3^ Among the available techniques, a relatively simple approach to interrogate the strength and selectivity of G4-ligand interactions is using oligonucleotides modified site-specifically with fluorescent nucleobases.^4^ One such nucleobase is 5-furyl-2′-deoxyuridine (5FU), originally developed by Tor and coworkers for the detection of abasic sites in duplexes.^5^5FU is a viscosity-sensitive fluorescent molecular probe that can undergo rotational motion, leading to twisted excited states. As a result, its fluorescence intensity (FI) is highly dependent on its local environment: low intensity in unconstrained environments (due to internal rotation) and high intensity in sterically hindered/viscous settings, where structural rigidification decreases the contribution of non-radiative decay pathways.^6^ Previously, 5FU and derivatives were embedded in G4 loops to study ligand-induced structural changes, including aptamer binding through a turn-on mechanism (Fig. 1A),^7^ G4-topology shifts,^8,9^ and ligand binding *via* turn-off mechanisms (Fig. 1B).^10^ Different G4-binding ligands stack on terminal G-tetrads, displacing natural capping bases. We hypothesise that peptide-based ligands, due to their bulky nature, can fully displace the capping residues without allowing them to π-stack above the ligand, leading to a net turn-on in fluorescence as a result of displacement from a highly quenching environment to a less quenching environment (Fig. 1c). To this end, here we describe the use of 5FU (Fig. 1d) as a fluorescent reporter in the capping regions for monitoring the interactions of a model G4 and top stacking ligands, in particular peptide-based ligands. For our model system, we selected the sequence T95-2T G4 in complex with RNA helicase associated with AU-rich element (RHAU) peptide.^3*a*^ Upon observation of the reported NMR structures for the bound (PDB: 2N21) and unbound complex (PDB: 2LK7) (Fig. S23), we noticed a significant change in the environment for capping dT 1,2 and 18.^11^ When unbound, the dTs stack on top of the terminal tetrads of the G4, engaging in a stable interaction. Upon peptide binding, these dT-caps are expelled into the solvent due to the steric clash with the alpha-helix of the peptide. We hypothesised that the change would be sufficient to accurately discriminate between bound and unbound states. To this end, we incorporated 5FU into all the different dT positions of T95-2T (Table 1) to investigate the changes in fluorescence intensity for each modified position.

**Fig. 1:**
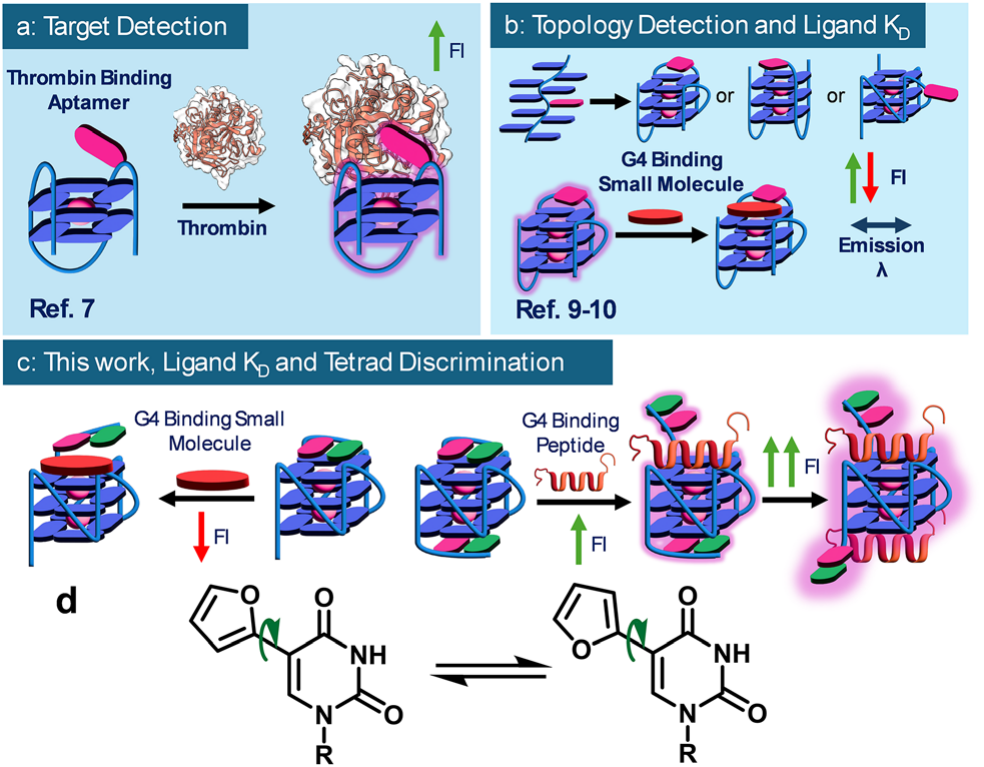
Previous work with 5FU derivatives in G-quadruplex systems, (a) turn-on target detection through 5FU modified aptamers, (b) detection of different topologies in human telomere sequence and ligand *K*_d_ determination, (c) this work, (d) 5-furyl-2′-deoxyuridine (5FU).

**Table 1:** Sequence and fluorescence behaviour of the oligonucleotides used

ODN	Sequence (5′ → 3′)	Fluorescence
T95-2T	TTGGGTGGGTGGGTGGGT	N/A
cKIT2	CGGGCGGGCGCTAGGGT	N/A
ODN-1	(5FU)TGGGTGGGTGGGTGGGT	Turn-on
ODN-2	T(5FU)GGGTGGGTGGGTGGGT	Turn-on
ODN-3	TTGGG(5FU)GGGTGGGTGGGT	No change/turn-off
ODN-4	TTGGGTGGG(5FU)GGGTGGGT	No change/turn-off
ODN-5	TTGGGTGGGTGGG(5FU)GGGT	No change/turn-off
ODN-6	TTGGGTGGGTGGGTGGG(5FU)	Turn-on
ODN-7	GGT(5FU)GGTGTGGTTGG	No change
ODN-8	T(5FU)GGGTGGGTGGGTGGG(5FU)T	Turn-on
ODN-9	(5FU)GGGCGGGCGCTAGGGAGGGT	Turn-on
ODN-10	TGGGCGGGCGCTAGGGAGGG(5FU)T	Turn-on
ODN-11	(5FU)GGGCGGGCGCTAGGGAGGG(5FU)T	Turn-on
ODN-12	AAGGGAAGGG(5FU)A	Turn-on
ODN-13	(5FU)GGGGT	Turn-on

Of particular interest for our study are ODN-2 and ODN-6, as these sequences are characterised by a furan-modified base close to the 3′- and at the 5′-positions respectively, and, therefore, at the two different and opposite tetrads of the G4 structure (top and bottom), which could potentially indicate the preferred site of binding for RHAU23. Titration studies for both sequences show an increase in fluorescence intensity of approximately threefold, for both the modified ODN-2 and ODN-6 as the amount of titrated RHAU23 increases (Fig. 2a and Fig. S18f in SI). The same pattern, with a minor twofold increase of fluorescence intensity, was observed for ODN-1 (Fig. S18a). By plotting the fluorescence intensity maximum (at *λ* = 435nm) for each ODN of interest upon addition of the peptide, we were able to extrapolate *K*_d_ values of 0.33 ± 0.02 µM and 1.5 ± 0.1 µM for RHAU23 when bound to 5′ and 3′ tetrads, respectively, demonstrating a net tetrad preference for the peptide. The overall *K*_d_ value is in line with what was calculated through Native PAGE analysis.^12^ Four different negative controls were considered for our experiments: ODN-3, ODN-4, and ODN-5, which contain 5FU in three different loop positions, and therefore should experience minor perturbation in the environment; an anti-parallel control derived from the thrombin-binding aptamer (TBA), modified in the loops with 5FU, which should not interact with RHAU23 at the experimental conditions used for the titration experiment (ODN-7). The modification in position 4 was chosen as the dT residue (similarly to the terminal dT residues of T95-2T) stacks on top of the TBA tetrad, thus maintaining a similar design when varying the G4-topology from parallel to antiparallel. All these negative controls were characterised by a negligible variation in FI upon peptide binding (Fig. 2c for ODN-4, Fig. S18c for ODN-3, Fig. S18e for ODN-5 and Fig. S18g for ODN-7). While for the TBA model ODN-7, the little negative variation is likely due to a minor dilution effect due to the titration, for the loop-substituted ODN-3, ODN-4 and ODN-5, a minor fluorescence turn-off was recorded, in agreement with the previous reports utilising 5FU modification on the G4 loops rather than in the capping regions.^8,9^ To rationalise the observed fluorescence turn-on upon RHAU23 binding to ODN-1, ODN-2, and ODN-6, we propose a mechanism where the alpha-helix of the peptide displaces 5FU, while stacking directly on the terminal G-tetrads. Such steric displacement would be expected to disrupt π-stacking, increase fluctuations in the inter-ring dihedral angle between the two aromatic components of 5FU (furan and thymine) and therefore lower its emission quantum yield.^6*a*^ In our experiments, we observe the opposite effect, an increase in fluorescence emission. We hypothesise that when 5FU is stacked on the terminal tetrad, it is subject to strong quenching by adjacent guanines, the most readily oxidizable nucleobase.^13*a*^ This is consistent with the behaviour of other nucleoside analogues such as 2-aminopurine, where static quenching results in lower fluorescence emission, which is regained upon displacement towards extra-helical environments.^13*b*^ In this scenario, excited-state 5FU functions as an electron acceptor while guanines act as the donor, allowing photoinduced electron transfer quenching.^14^ Therefore, upon peptide binding and ejection of the base into a less-guanine-rich environment, the fluorescence is partially restored, resulting in a turn-on effect. This shift from a highly quenched “off” state to a “partially” emissive on state has been described for similar nucleobases in duplex DNA by Tor and co-workers.^5^ This fluorescence response is particularly observed when 5FU is positioned in the capping regions (ODN-1, ODN-2, and ODN-6; see SI Section S4 and Fig. 2), and it is consistent with the structural model. Of note is the higher increase in fluorescence for ODN-2 and ODN-6 compared to ODN-1 upon peptide binding, reflecting the position of the 5FU residue only partially stacking on top of the 5′ tetrad (functioning as an additional control in supporting the proposed fluorescence turn-on mechanism). The modest bathochromic shift in the emission maxima observed is a further evidence for the mechanism proposed, as it suggests that 5FU, upon peptide binding, is moved to a relatively more solvent-exposed and polar environment (in line with polarity sensitivity of the probe).^5^ Having established the turn-on fluorescence response for RHAU23, we decided to test the fluorescence response with well-characterised small-molecule G4 ligands, using ODN-2 as a model system. For this purpose, we tested the ligands BRACO-19, TMPyP4, and Phen-DC3, all of which are known to bind G-quadruplexes with multiple binding modes, principally G-tetrad stacking.^15–17^ Interestingly, and in contrast to the increase in fluorescence caused by RHAU23, the titration of ODN-2 with BRACO-19 resulted in progressive quenching of FI, and could possibly indicate a different interaction with the DNA and the capping residue (Fig. 2d). This reduction was accompanied by a modest blue-shift in the emission maximum, indicating a different (presumably less polar) environment for 5FU. A similar turn-off behaviour was observed with TMPyP4 and Phen-DC3 (Fig. S19). Structural insights from the NMR structure of BRACO-19, with a bimolecular parallel-stranded human telomeric G4 (PDB: 3CE5, Fig. S24),^17^ could support a model in which the ligand intercalates between the terminal G-tetrad and the capping base π-stacking on the organic ligand. This arrangement both restricts the rotation of the 5FU and brings it into proximity with the more apolar π-systems, acting as fluorescence quenchers, and can help explain the fluorescence turn-off and the hypsochromic shift observed. Other structural studies reveal that TMPyP4 typically π-stacks on the terminal tetrads, which could, in principle, favour π-stacking of the 5FU on top of the ligand (PDB: 2HRI),^18^ while Phen-DC3 can also intercalate between G-tetrads (PDB: 7Z9L).^15^ Both binding modes probably contribute to the quenching of the 5FU probe. Reverse displacement experiments (ligand titration with subsequent titration with RHAU23) were also performed, which led to a partial restoration of fluorescence and red-shifted emission band, suggesting that the peptide can displace the ligand and eject the 5FU into a less quenched and more polar environment (Fig. S20). This reversible modulation of fluorescence intensity reinforces the interpretation that these ligands compete for overlapping binding sites but differ in how they interact with the capping base. While fluorescence responses alone cannot be used as definitive proof of a shared binding mode, previous studies demonstrate that emission behaviour can, in certain systems, act as a reliable indicator of DNA interaction modes. For example, DAPI exhibits bright blue emission when bound in the AT-rich minor groove, but its fluorescence is quenched and its lifetime shortened when intercalated into GC-rich sequences.^19^ Similarly, the two enantiomers of the ruthenium polypyridyl complex (e.g Δ- and Λ-[Ru(phen)_2_dppz]^2+^) are characterised by distinct binding modes to DNA, demonstrated through X-ray crystallography, which are reflected in differences in quantum yield and excited-state lifetime.^20^ While our findings do not allow such a definitive conclusion in this case, these analogies illustrate why monitoring FI responses can still provide valuable qualitative insights into ligand–DNA interactions, for ligands characterised by similar, planar geometry. Therefore, in this particular context and using similar model systems, the turn-on effect observed with peptide-based ligands may indicate a possible, distinctive feature of this class of molecules. We further investigated tetrad specificity using a dual-labelled construct (ODN-8), in which 5FU was incorporated at both the 5′ and 3′ ends of the G4. Fluorescence titrations with RHAU23 yielded a biphasic response, consistent with sequential peptide binding, first to the 5′ tetrad, followed by the 3′ tetrad at higher concentrations. The obtained binding constants calculated from ODN-8 are in line with what was previously obtained using ODN-2 and ODN-6 separately (Fig. 3a) and backed up by NMR structural data (PDB: 2N21), which show preferential peptide interaction at the 5′ tetrad. The trend is also quantitatively supported by the distinct dissociation constants obtained from singly labelled constructs (0.33 ± 0.02 µM for 5′; 1.5 ± 0.10 µM for 3′). To demonstrate the applicability of the concept to other G4s, we applied our method to c-KIT G4, a parallel-stranded structure that is characterised by a loop-stacked adenosine over the 5′ tetrad, as revealed by NMR data (PDB: 7NWD).^21^ To investigate tetrad accessibility, we site-specifically incorporated 5FU at either the 5′ (ODN-9) or 3′ (ODN-10) ends. Fluorescence titrations with RHAU23 (Fig. S18i and j) revealed a turn-on response in both cases, with a significantly greater intensity increase observed for the 3′-labelled construct, in contrast to what was previously observed for T95-2T G4-sequence. This result suggests that the loop architecture sterically hinders the peptide access to the 5′ tetrad (1.3 ± 0.10 µM), favouring the binding toward the 3′ face preferentially (0.8 ± 0.10 µM) (Fig. 3b). When both ends were simultaneously modified (ODN-11), the overall fluorescence increase upon peptide saturation was intermediate and (Fig. S18k), consistently with independent contributions from each tetrad. Although the relative preference of the peptide for the two tetrads prevented accurate *K*_d_ calculation using a single oligonucleotide (due to comparable *K*_d_ values causing overlapping titration curves), this was feasible for ODN-8, where sufficiently distinct *K*_d_ values allowed individual quantification. These findings demonstrate the ability of 5FU-probes to resolve tetrad-specific accessibility modulated by sequence-dependent structural features. The result obtained with ODN-8 was confirmed using CD titration, monitoring the decrease of the maximum at 264nm upon peptide addition (Fig. S23). To evaluate the applicability of the method to multimeric G4 DNAs, two additional constructs were prepared: A bimolecular G4 based on PDB: 8X1V,^22^ where the capping A residue is substituted with a 5FU (ODN-12), and a tetramolecular G4 based on PDB: 1O0K,^23^ where the capping T is substituted (ODN-13). CD spectroscopy indicated potassium-induced folding into a parallel G4 for both sequences (Fig. S16). 5FU insertion allowed for the calculation of a *K*_d_ in both cases (0.75 ± 0.07 µM for ODN-13) and, notably, for the bimolecular ODN-12, showed two consecutive binding events (0.61 ± 0.03 µM and 1.4 ± 0.03 µM, respectively), pointing again towards peptide binding to the two distinct tetrads (Fig. S18m and l and Fig. S22). However, without independent localisation of 5FU to a defined tetrad, assigning tetrad-specific *K*_d_ values remains challenging without the use of complementary high-resolution methods, such as NMR spectroscopy. Table S3 in SI summarises the *K*_d_ values obtained in the work. To study the effect of our cellular probe on a more biologically relevant set-up, a fluorescence titration with the doubly modified ODN-8 was performed in molecular crowding conditions (addition of 20% PEG600). Such conditions not only mimic the excluded-volume effects of the cellular environment, but also increase solution viscosity, both of which are known to influence nucleic acid structure and ligand-binding.^24^ In our case, crowding led to an overall threefold FI increase (Fig. S21), likely due to the higher viscosity of the environment, indicating that the probe remains functional, showing FI under these conditions. Results suggest that binding parameters can be extrapolated using lower quantities, under conditions that mimic cellular environment.

**Fig. 2:**
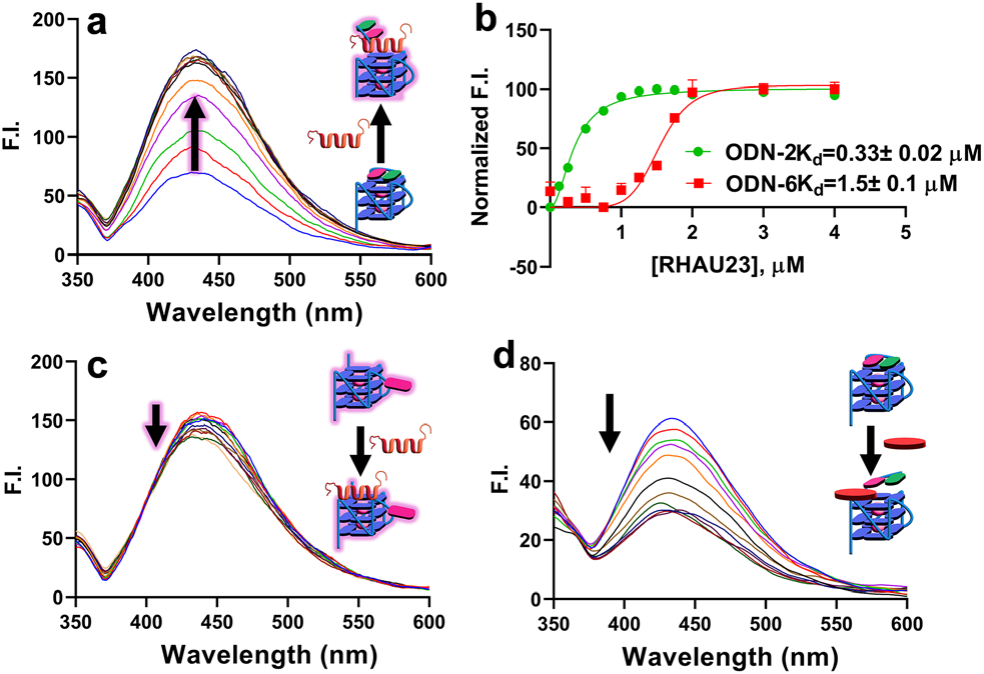
Fluorescence Spectra of ODN-2 when titrated with increasing eq. of RHAU23, (b) Normalised fluorescence intensity against concentration of RHAU23 for ODN-2 and ODN-6 for the determination of *K*_d_*via* the Hill equation, (c) fluorescence Intensity of control experiment ODN-4 when titrated with increasing eq. of RHAU23, (d) fluorescence intensity of ODN-2 when titrated with increasing eq. of BRACO-19.

**Fig. 3:**
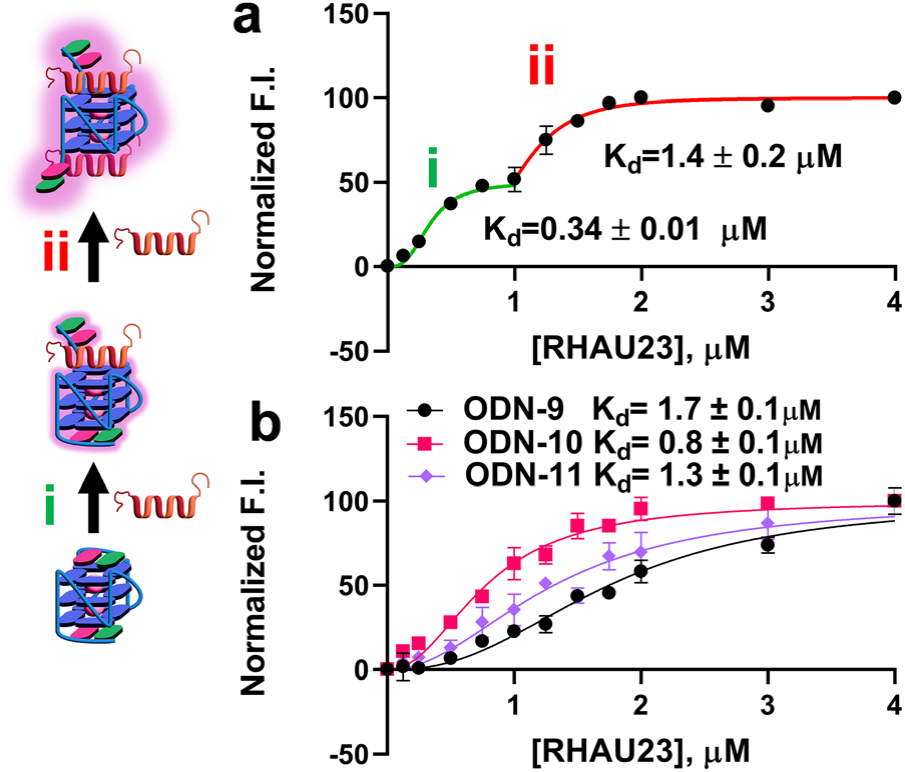
*K*_d_ determination from fluorescence titration data for T95-2T and c-KIT model. (a) Normalised fluorescence intensity against concentration of RHAU23 for doubly modified ODN-8; (b) normalised fluorescence intensity against concentration of RHAU23 for c-KIT analogues ODN-9, -10 and -11.

## Conclusions

Here, we report how peptide-based ligands can effectively displace the T nucleobase at terminal positions, increasing FI emission using 5FU-based probes. This enables *K*_d_ calculation for specific tetrads within the same G4. When *K*_d_ values differ sufficiently, a single doubly modified ODN permits calculation of both constants. These findings establish 5FU-labelled probes as powerful tools for resolving binding orientation, offering an accessible alternative and complement to high-resolution structural techniques. In the future, we plan to use structural 5FU analogues with distinct fluorescence excitation/emission parameters, which would, in principle, enable independent and simultaneous study of peptide binding events on both tetrads of relevant G4s with higher precision (currently one of the main limitations to the technique). We foresee expanding the method to study G4-RNA structures (in view of the similarities with parallel DNA G4s) and more complex protein-DNA models.

## Conflicts of interest

There are no conflicts to declare.

## Supplementary Material

CB-007-D5CB00247H-s001

## Data Availability

All the data presented in this manuscript are available in the Supplementary information (SI). Supplementary information: raw fluorescence data, procedures, visualisation and molecule characterisation. See DOI: https://doi.org/10.1039/d5cb00247h.
