# Supplementary material for: From loops to caps: discriminating peptide binding to distinct G-quadruplex tetrads using 5-furyl-2′-deoxyuridine fluorescent probes
Source: RSC Chem Biol. 2025 Nov 10;7(1):44–8. doi: 10.1039/d5cb00247h (PMC12631246; doi:10.1039/d5cb00247h)
Supplement: CB-007-D5CB00247H-s001 [file CB-007-D5CB00247H-s001.pdf]

Supplementary Information (SI) for RSC Chemical Biology.
This journal is © The Royal Society of Chemistry 2025

# From Loops to Caps: Discriminating Peptide Binding to Distinct G-Quadruplex Tetrads Using 5-Furyl-2'-Deoxyuridine Fluorescent Probes

Jack Barr,[a‡] Tayler D. Prieto Otoya,[a,b‡] Christine Cardin,[b] and Enrico Cadoni[a*]

[a] Organic and Biomimetic Chemistry Research Group, Ghent University, Krijgslaan 281 S4, B-9000, Ghent, Belgium

[b] Department of Chemistry, University of Reading, Whiteknights, Reading, RG6 6AD, UK

‡ These authors equally contributed to the present work

*Correspondence: Enrico.Cadoni@UGent.be

## Contents

1. General Materials ........ 1
2. General Instruments ........ 1
3. General methods ........ 2
   HPLC Methods ........ 2
   Synthesis of 5-furyl-2'-deoxyuridine phosphoramidite ........ 2
   Synthesis of modified oligonucleotides ........ 3
   Synthesis of Peptides ........ 8
4. Fluorescence Titration Experiments ........ 10
5. Structures for rationalisation ........ 12

## 1. General Materials

Reagents for the synthesis of the modified ODNs (coupling and capping solutions, phosphoramidite building blocks and Glen-Pak columns for standard ODN purification and desalting), were obtained from Glen Research. All other reagents were purchased from Sigma-Aldrich, Fluka, Acros Organics, TCI Europe or Fluorochem and used without further purification. Unmodified ODN sequences were obtained from Integrated DNA Technologies (Leuven, Belgium). All HPLC-grade solvents were purchased from Chemlab nv. Deuterated solvents for NMR analysis were purchased from Euroisotope.

## 2. General Instruments

Automated DNA synthesis was carried out on an ABI Expedite 8909.

Oligonucleotide purification was carried out with GLENPAK or SEPPAK cartridges.

Peptide synthesis was carried out on an INTAVIS MultiPep Synthesiser

HPLC (analytical) analyses and purifications were performed on either an Agilent 1100 or 1200 series instrument, equipped with Waters X-Bridge BEH C18 XP Column (130 Å, 2.5 µm, 4.6 mm X 50 mm), **method A**: Flow rate 0.8 mL/min, from 100 % A and 0 % B to 70 % A and 30 % B over 13 min. Solvent A: 5 % acetonitrile, 95% Triethylammonium acetate buffer 0.1M. Solvent B: Acetonitrile; Phenomenex Kinetex C18 100 Å column (150 × 4.6 mm, 5 µm at 35 °C), **method B**: Flow rate of 1.5ml/min, using a gradient from 100%A (mQ water + 0.1% Formic Acid) to 100 % B ($CH_3CN$) over 15 minutes; Luna C18(2) column (5 µm, 100 Å, 250x10 mm, 40°C), **method C**: flow rate of 4.0 ml/min, using gradient from 100%A (mQ water + 0.1% TFA) to 100 % B ($CH_3CN$) over 30 minutes.

MS analysis of the synthesised oligonucleotides, stapled oligonucleotides and DPCs was performed on a Thermo Fisher Scientific Q Exactive Plus Orbitrap Mass Spectrometer or by MALDI-TOF using an Applied Biosystems – 4800 Plus MALDI TOF/TOF™ Analyzer

HPLC-MS characterisation of the peptides used in this study was performed on a Agilent 1100 Series instrument equipped with a Phenomenex Kinetex C18 100 Å column (150 × 4.6 mm, 5 µm at 35 °C) connected to an ESMSD type VL mass detector (quadrupole ion trap mass spectrometer), using HPLC **Method B** conditions (*vide supra*).

$^1H$, $^{13}C$ and $^{31}P$ NMR spectra of each synthesised building block were recorded at room temperature on a Bruker Avance 300 or 400 MHz.

Circular Dichroism experiments were conducted on a AVIV Biomedical Inc. (Lakewood, NJ, USA) Circular Dichroism Spectrometer 410.

Concentration determinations of ODNs were performed using a Thermo Fischer Nanodrop spectrometer taking the average of 2 measurements at room temperature, determining the absorbance at 260 nm for DNA. The molar extinction coefficient for each ODN was calculated according to the literature.[1]

Normal phase purifications were performed on a Grace Reveleris iES flash chromatography instrument, containing a class II, ImW-635 nm laser.

Visualisation and adaptation of solution structures were performed with ChimeraX software with data downloaded from the Protein Data Bank (PDB).

Fluorescence titration experiments were performed on a Varian Cary Eclipse Instrument equipped with a six-cell thermostatic cell holder and temperature controller.

## 3. General methods

### Synthesis of 5-furyl-2'-deoxyuridine phosphoramidite

The synthesis of the phosphoramidite for incorporation of **5FU** was carried out according to previous literature reports (**Figure S1**).[2]

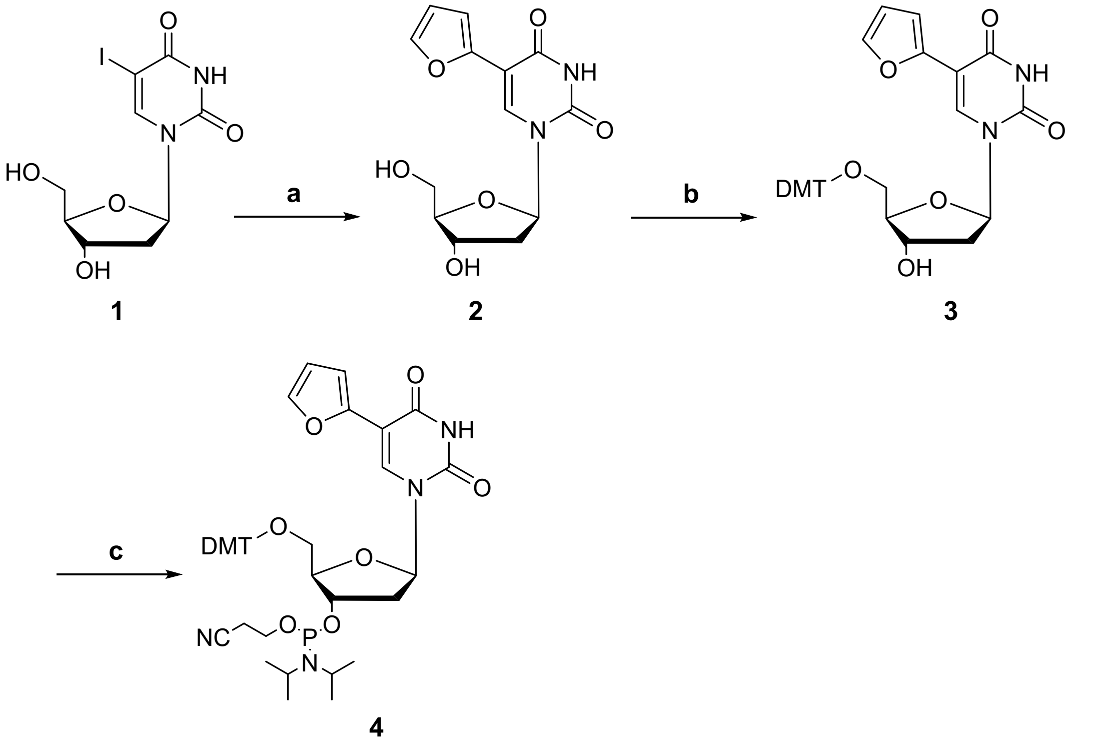


Figure S 1. Synthetic scheme towards phosphoramidite **4 (5FU**) Conditions: a) $PdCl_2(PPh)_2$ (0.02 eq), 2-(tributylstannyl)furan (1.8 eq), Dioxane (Anh.), 90 °C, 70.2%; b) DMTCl (1.5 equiv), pyridine, RT, overnight, 73%; c) $(iPr_2N)_2(NCCH_2CH_2O)PCl$, diisopropylammonium tetrazolide (1.5 equiv), $CH_2Cl_2$, 0°C to RT, Overnight 79%.

## Synthesis of modified oligonucleotides

Oligonucleotide solid phase synthesis was performed on an Applied Biosystems Expedite 8910 oligonucleotide synthesiser. All the oligonucleotides were synthesised at 1 micromolar scale, DMT-on, using standard coupling procedures, using a 3'-pre-loaded resin containing the desired nucleobase. For the insertion of modified bases, the oligonucleotides were synthesised DMT-off, the column removed, and a manual coupling procedure was performed as follows: the desired phosphoramidite was dissolved in acetonitrile at a final concentration of 0.1 M, under argon. 0.4 mL of activator solution (containing 0.25M ETT in dry MeCN) is taken with a syringe and loaded in the column. 0.5 mL of the phosphoramidite solution is taken with a new syringe and connected to the opposite side of the column. Small portions of each solution are alternately pushed through the column over 20 minutes. After this time, the solutions were disposed of, the column was rinsed with MeCN and reinstalled into the synthesiser and the automated synthesis was resumed for the rest of the sequence.

Oligonucleotides were then cleaved and deprotected using 1 mL of 30% $NH_4OH$ solution at 55 °C overnight. Following this the oligonucleotide was purified on a DMT selective cartridge according to the manufacturer's instructions and used without further.

ODN were analysed by Orbitrap-MS or MALDI-TOF with the following matrix, prepared fresh and applied matrix first then ODN sample:

**Matrix**: a 8:1 ratio the following solutions: 50 mg/mL 3-hydroxypicolinic acid (HPA) in mQ water: MeCN (1:1, solution 1) + 50 mg/mL ammonium citrate in mQ water

Purity of ODN samples was checked by HPLC (**Method A**)

*Table S1. Oligonucleotides synthesised in this study. Modifications to the wild type sequence are in bold*

| ODN | Sequence (5'-3') | MW | Fluorescence | Yield %* |
|---|---|---|---|---|
| **T95-2T†** | TTGGGTGGGTGGGTGGGT | 5713.8 | N/A | NA |
| **cKIT 2** | CGGGCGGGCGCTAGGGAGGGT | 6688.3 | N/A | |
| **ODN-1** | **(5FU)**TGGGTGGGTGGGTGGGT | 5765.5 | Turn-on | 17.9 |
| **ODN-2** | T**(5FU)**GGGTGGGTGGGTGGGT | 5765.5 | Turn-on | 18.6 |
| **ODN-3** | TTGGG**(5FU)**GGGTGGGTGGGT | 5765.5 | No change / turn-off | 1.5 |
| **ODN-4** | TTGGGTGGG**(5FU)**GGGTGGGT | 5765.5 | No change / turn-off | 1.5 |
| **ODN-5** | TTGGGTGGGTGGG**(5FU)**GGGT | 5765.5 | No change / turn-off | 1.9 |
| **ODN-6** | TTGGGTGGGTGGGTGGG**(5FU)** | 5765.5 | Turn-on | 1.6 |
| **ODN-7** | GGT**(5FU)**GGTGTGGTTGG | 4778.2 | No change | 9.5 |
| **ODN-8** | T**(5FU)**GGGTGGGTGGGTGGG**(5FU)**T | 6069.9 | Turn-on | 9.9 |
| **ODN-9** | **(5FU)**GGGCGGGCGCTAGGGAGGGT | 6676.3 | Turn-on | 50.2 |
| **ODN-10** | TGGGCGGGCGCTAGGGAGGG**(5FU)**T | 6980.4 | Turn-on | 51.7 |
| **ODN-11** | **(5FU)**GGGCGGGCGCTAGGGAGGG**(5FU)**T | 7032.5 | Turn-on | 42.5 |
| **ODN-12** | AAGGGAAGGG(**5FU**)A | | Turn-on | 40.2 |
| **ODN-13** | (**5FU**)GGGGT | 1915.2 | Turn-on | 38.7 |

* Yields were calculated with respect to synthesis scale of 1 µmol by determining the absorbance at 260 nm (average of 2 measurements at room temperature) of a representative sample of known volume using a nanodrop 1 spectrometer. The molar extinction coefficient for DNA was calculated according to the literature.[1]

† Unmodified ODNs were purchased and therefore no yield was calculated

## ODN-1

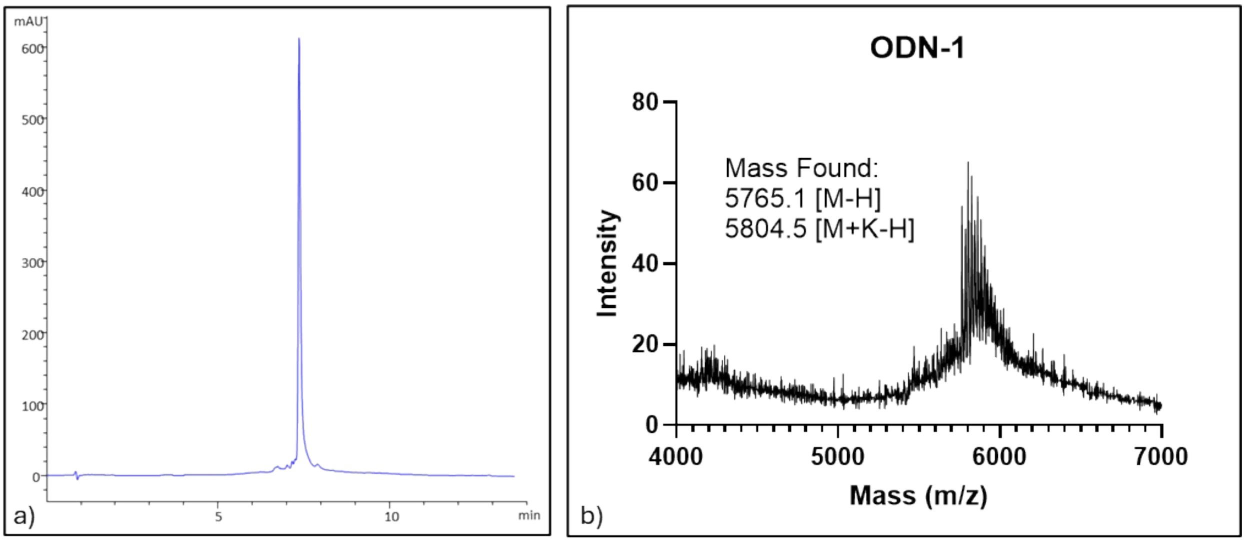


Figure S 2a: HPLC Chromatogram of **ODN-1** at 260 nm wavelength**, b:** MALDI-TOF spectrum of **ODN-1**. Calculated MW: 5765.5, Found: 5765.1 [M-H]$^-$ and 5804.5 [M-H+K]$^-$

## ODN-2

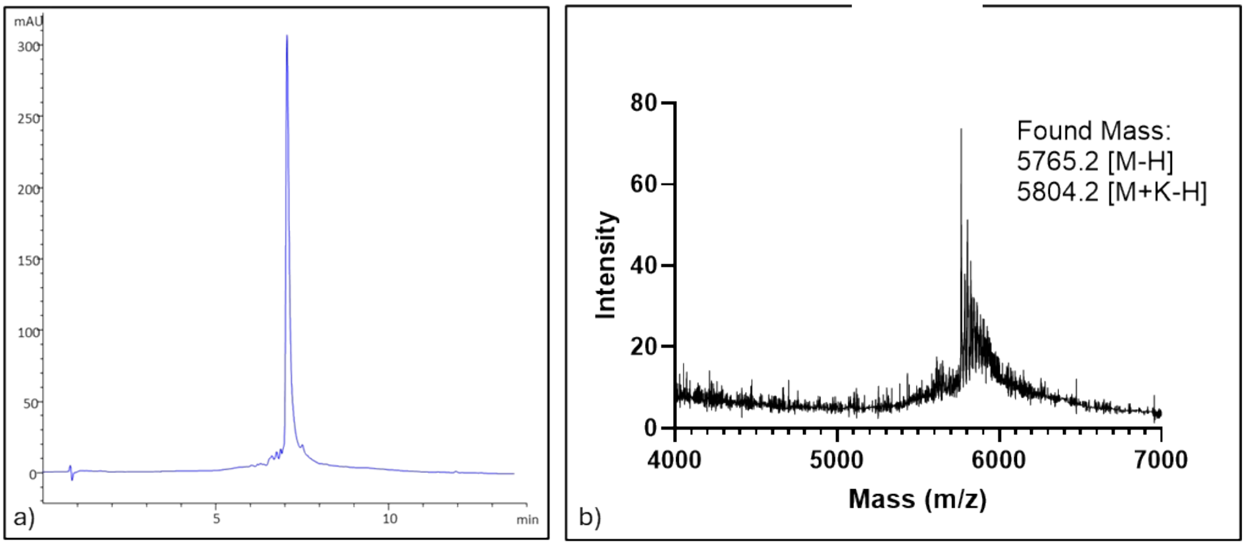


Figure S 3a: *HPLC Chromatogram of **ODN-2** at 260 nm wavelength, b: MALDI-TOF spectrum of **ODN-2**. Calculated MW: 5765.5, Found: 5765.2 [M-H]$^-$ and 5804.2 [M-H+K]$^-$*

## ODN-3

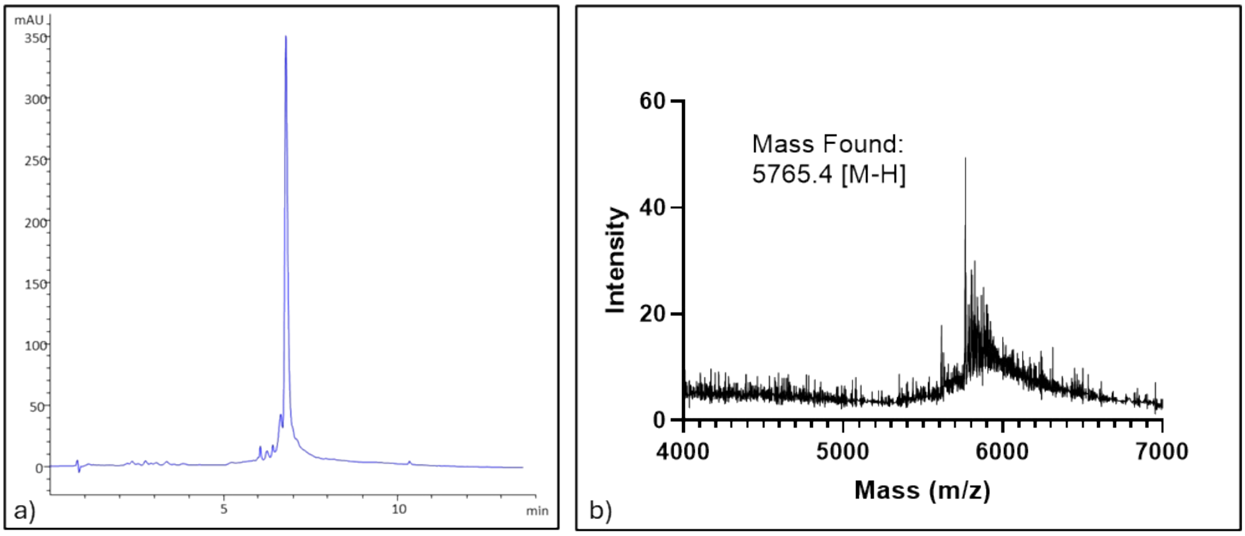


Figure S 4a: *HPLC Chromatogram of **ODN-3** at 260 nm wavelength, b: MALDI-TOF spectrum of **ODN-3**. Calculated MW: 5765.5, Found: 5765.4 $[M-H]^-$*

## ODN-4

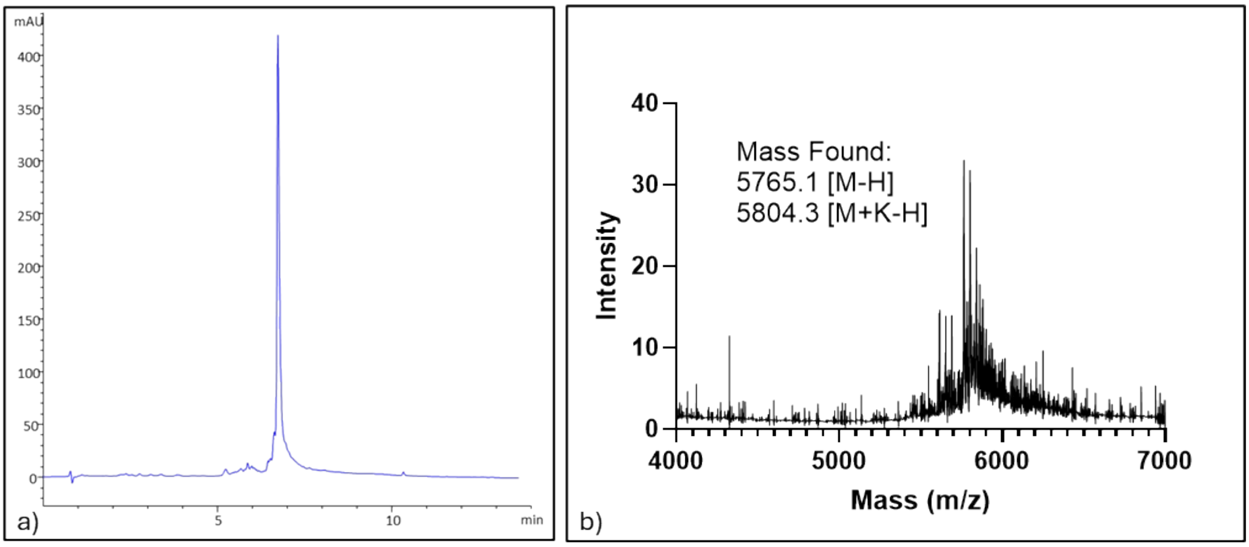


Figure S 5a: *HPLC Chromatogram of **ODN-4** at 260 nm wavelength, b: MALDI-TOF spectrum of **ODN-4**. Calculated MW: 5765.5, Found: 5765.4 $[M-H]^-$*

## ODN-5

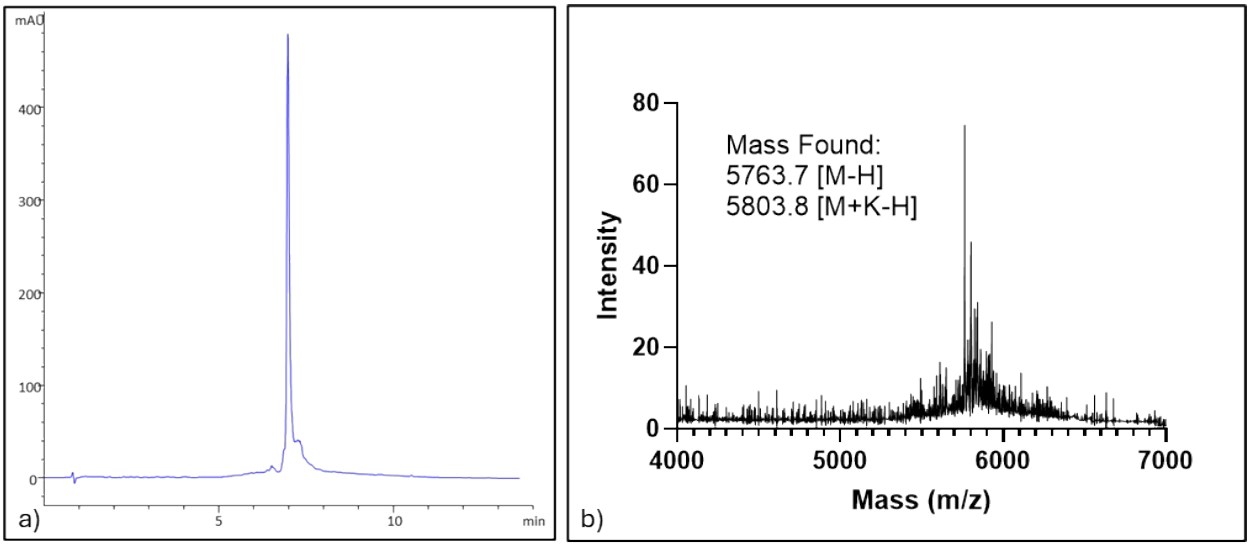


Figure S 6a. *HPLC Chromatogram of **ODN-5** at 260 nm wavelength, b: MALDI-TOF spectrum of **ODN-5**. Calculated MW: 5765.5, Found: 5765.4* $[M-H]^-$

## ODN-6

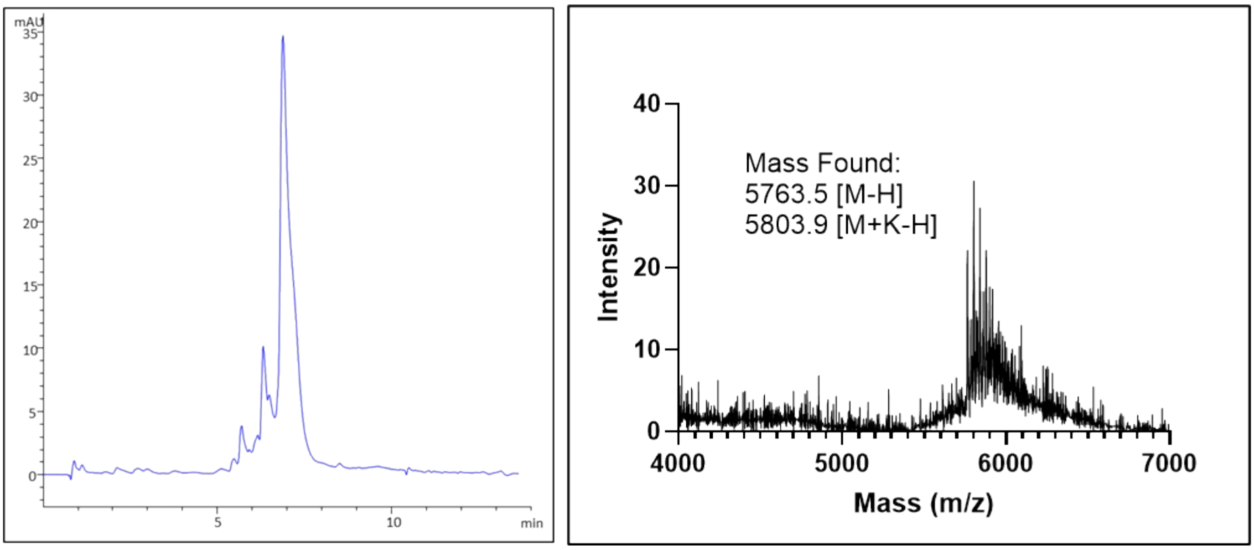


Figure S 7a*: HPLC Chromatogram of **ODN-6** at 260 nm wavelength, b: MALDI-TOF spectrum of **ODN-6**. Calculated MW: 5765.5, Found: 5765.4* $[M-H]^-$

## ODN-7

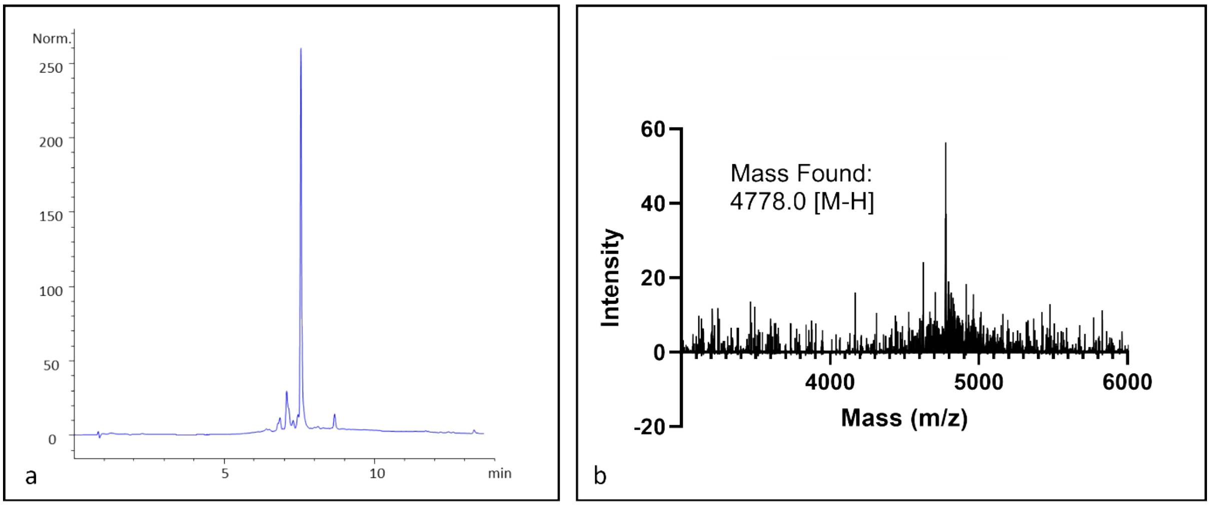


Figure S 8a*: HPLC Chromatogram of* ***ODN-7*** *at 260 nm wavelength, b: MALDI-TOF spectrum of* ***ODN-7***. *Calculated MW: 4778.2, Found: 4778.0* $[M\text{-}H]^-$

## ODN-8

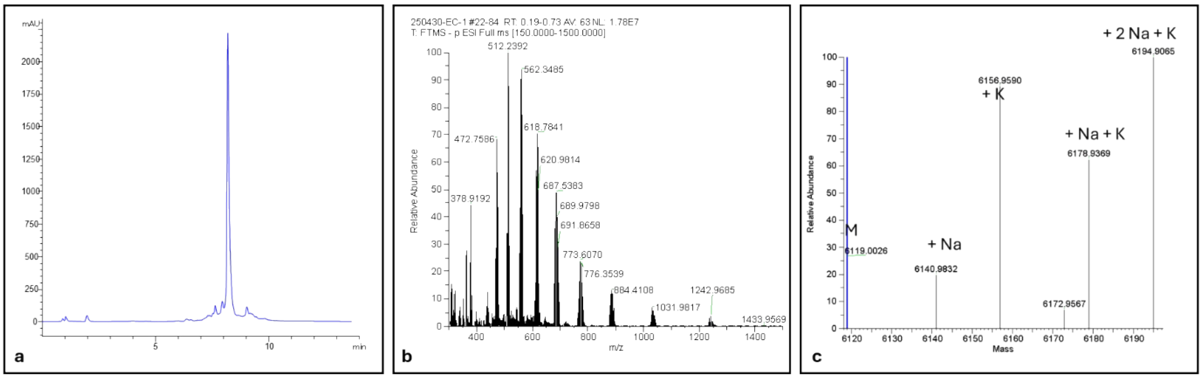


Figure S 9a*: HPLC Chromatogram of* ***ODN-8*** *at 260 nm wavelength, b: mass spectrum* ***ODN-8,*** *c: Calculated MW: 6121.7 Found: 6119.0 [M-H], 6141.0 [M-H + Na]-, 6157.0 [M-H +K]-, 6179.0 [M-H +Na +K]-, 6195.0 [M-H +2Na +K]-.*

## ODN-9

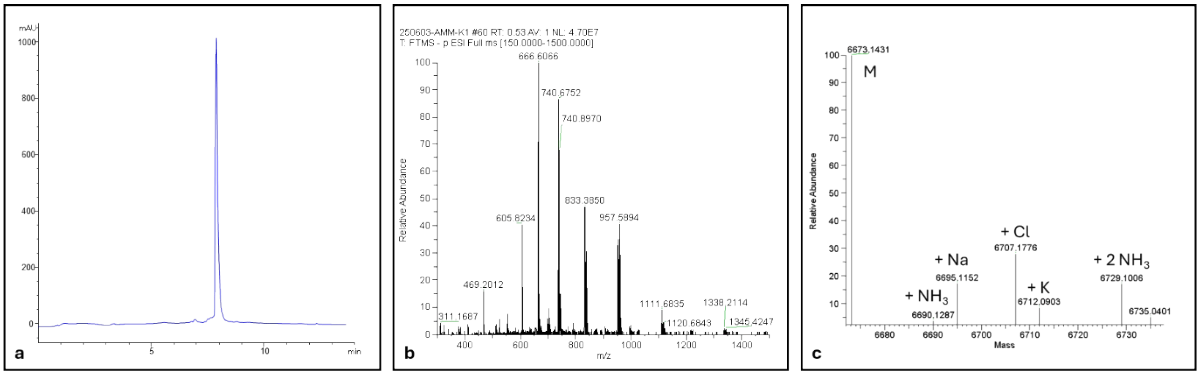


Figure S 10a*: HPLC Chromatogram of* ***ODN-9*** *at 260 nm wavelength, b: mass spectrum* ***ODN-9,*** *c: Calculated MW: 6676.3 Found: 6673.1 [M-H], 6695.1 [M-H + Na]-, 6712.1[M-H +K]-, 6729.1 [M-H +2*$NH_3$*]-.*

## ODN-10

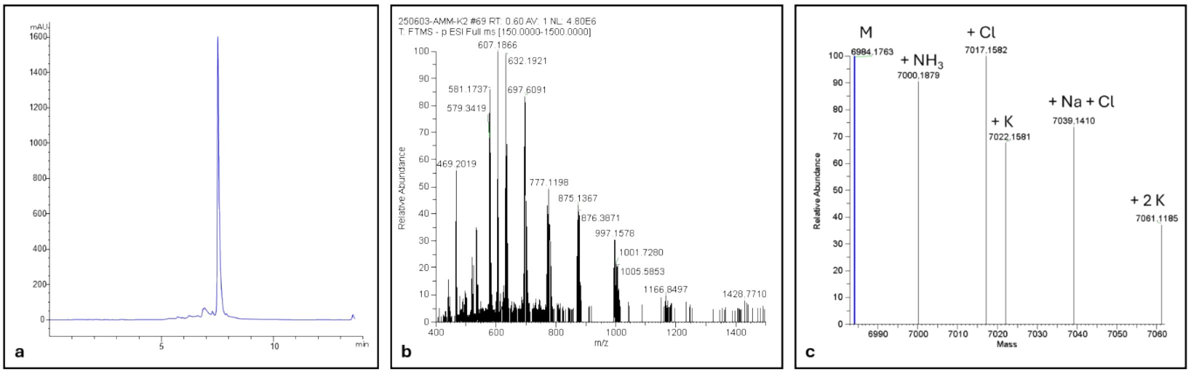


Figure S 11a: *HPLC Chromatogram of* ***ODN-10*** *at 260 nm wavelength, b: mass spectrum* ***ODN-10,*** *c: Calculated MW: 6980.4 Found: 6984.2 [M-H], 7000.2 [M-H + $NH_3$]-, 7022.2 [M-H +K]-, 7039.2 [M-H +Na +Cl]-]-, 7612.1 [M-H +2K]-.*

## ODN-11

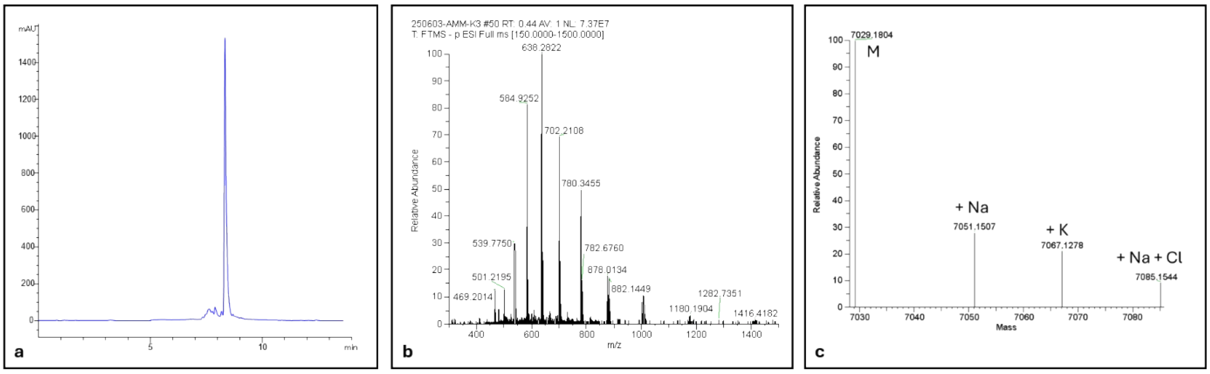


Figure S 12a: *HPLC Chromatogram of* ***ODN-11*** *at 260 nm wavelength, b: mass spectrum* ***ODN-11,*** *c: Calculated MW: 7032.5 Found: 7029.2 [M-H], 7051.2 [M-H + Na]-, 7067.2 [M-H +K]-, 7085.2.2 [M-H +Na +Cl]-]-*

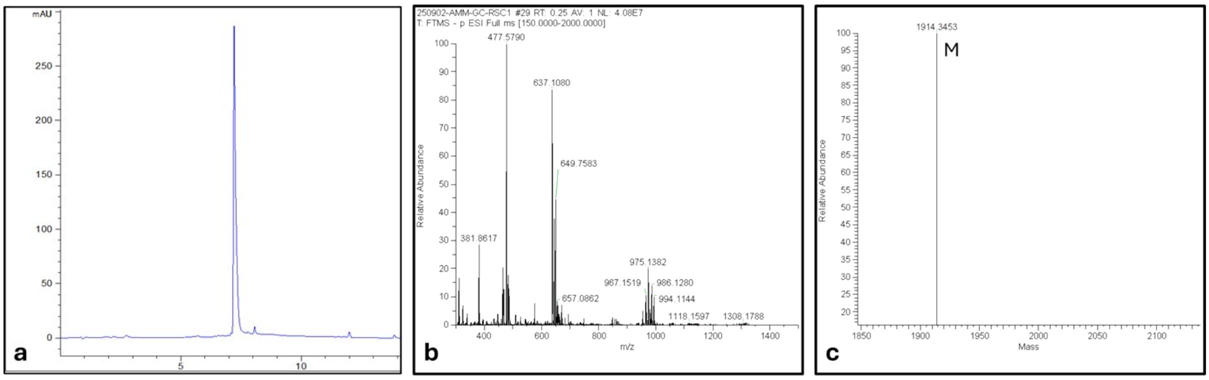


Figure S 13a: *HPLC Chromatogram of* ***ODN-12*** *at 260 nm wavelength, b: mass spectrum* ***ODN-12,*** *c: Calculated MW: 1915.2 Found: 1914.3 [M-H]*

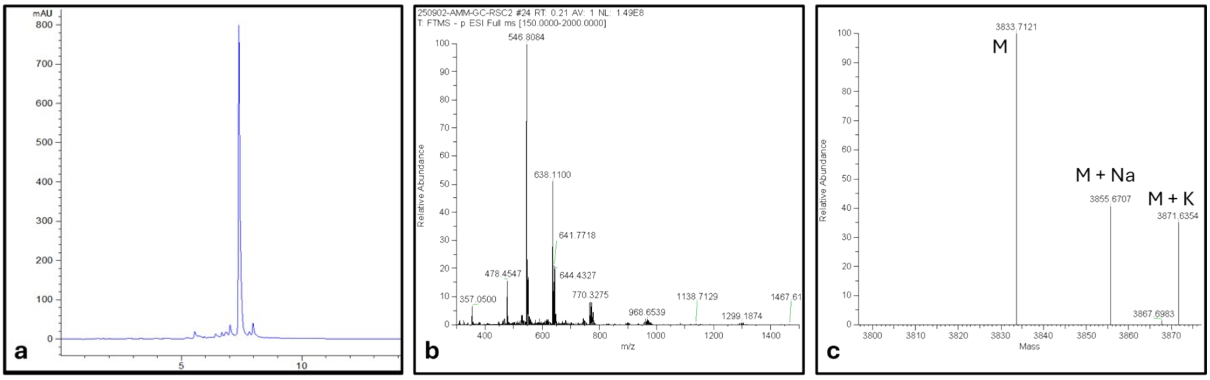


Figure S 14a*: HPLC Chromatogram of* ***ODN-13*** *at 260 nm wavelength, b: mass spectrum* ***ODN-13,*** *c: Calculated MW: 3835.4 Found: 3833.7 [M-H], 3855.7 [M-H + Na]-, 3871.6 [M-H +K]-.*

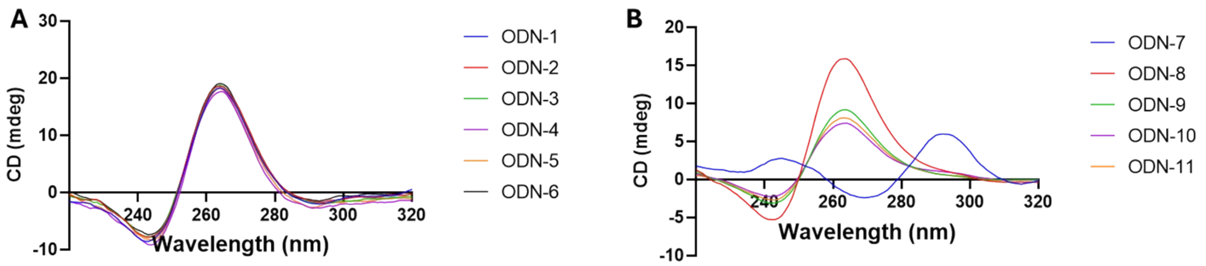


Figure S 15. CD spectra of the oligonucleotides synthesized in this study under the experimental conditions described in section 4 (KCl (70 mM) and $K_2HPO_4$ buffer (pH 7, 10 mM)), at a final DNA concentration of 5 μM. A. CD spectra of **ODN-1** to **ODN-6**; B. CD spectra of **ODN-7** to **ODN-11**.

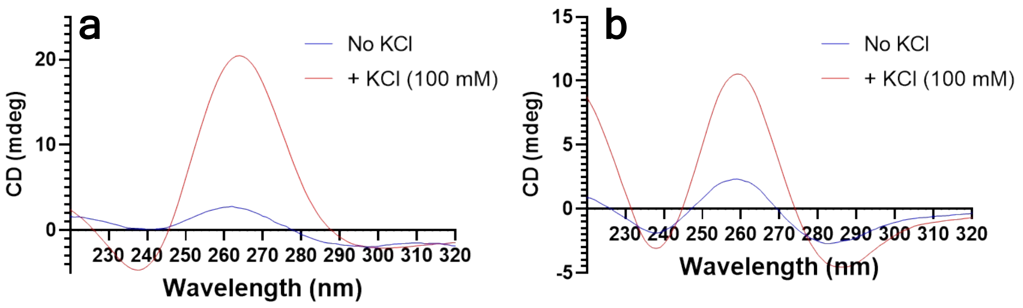


Figure S 16. CD spectra of the multimeric G4-folding oligonucleotides synthesized in this study, in Lithium phosphate buffer pH 7 (no KCl, blue curves) and the experimental fluorescence titration conditions described in section 4 (KCl (70 mM) and $K_2HPO_4$ buffer (pH 7, 10 mM), red curves), at a final DNA concentration of 5 μM. A) CD spectra of **ODN-12**; B) CD spectra of **ODN-13**.

## Synthesis of Peptides

Solid phase synthesis of peptides were synthesized according to standard Fmoc-based solid-phase peptide synthesis methodology, using a Rink amide AM resin (loading: 0.64 mmol/g) on an INTAVIS MultiPep Synthesiser, using HATU/DIPEA as coupling mixture (4 eq. Fmoc-AA-OH, 4 eq. HATU, 8 eq. DIPEA for each coupling), and each coupling step repeated twice for 30 minutes at room temperature. The Fmoc group removal was performed with a solution of 30% piperidine in DMF. After each coupling cycle, the resin was capped with a mixture containing 6.5% acetic acid in DMF (capping mixture). At the end of the synthesis, the Fmoc group was removed (vide supra) and the free amino group at the N-terminal position capped with the capping mixture.

The cleavage of the resin from the solid support and the deprotection of the AA protecting groups was performed using a cocktail composed of 1,2-EDT (2.5%), Water (5%), thioanisole (5%) and m-cresol (5%)in TFA (82.5%).

The purification of the crude peptide was performed according to general HPLC **method C** (*vide supra*)

The HPLC-MS analysis of the purified peptides were performed on a 1100 Agilent HPLC system equipped with a DAD, and connected with a Agilent ESI-single quadrupole MS detector. The separation (HPLC) was performed on a Phenomenex Kinetex C18 column, 100 Å (150 x 4.6 mm, 5 µm, at 35 °C) using **Method B.**

*Table S2. Peptide synthesised in this study.*

| ODN | Sequence (N→C) | MW | Yield %* |
|---|---|---|---|
| **P1** | Acetyl-HPGHLKGREIGMWYAKKQGQKNK-$CONH_2$ | 2734.2 | 22.0 |

### RHAU 23

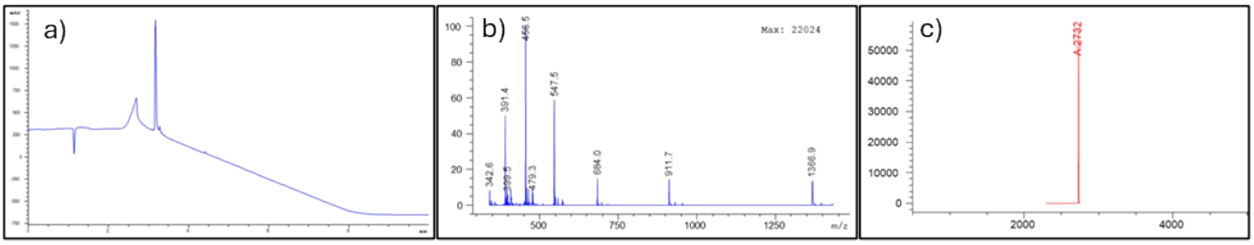


Figure S 17a*: HPLC Chromatogram of* ***RHAU 23*** *at 214 nm wavelength, b: MS spectrum of* ***RHAU 23****. Sequence (N to C terminus): Acetyl-HPGHLKGREIGMWYAKKQGQKNK-$CONH_2$ (Amide). Calculated MW: 2734.2 Da. Found: 1366.9 $[M+2H]^{2+}$, 911.7 $[M+3H]^{3+}$, 684.0 $[M+4H]^{4+}$, 547.5 $[M+5H]^{5+}$, 456.5 $[M+6H]^{6+}$, 391.4 $[M+7H]^{7+}$. c: Deconvoluted Mass of P2, Calculated MW: 2734.2 Da. Found: 2732*

## 4. Fluorescence Titration Experiments

Prior to titration ODNs were annealed at 1 μM with a salt complement of KCl (70 mM) and $K_2HPO_4$ buffer (pH 7, 10 mM). For the experiments in high molecular crowding conditions, the solution was supplemented with 20% PEG600. 1 mL of solution was then transferred to a quartz cuvette. The G4-binding ligand was titrated into the solution and mixed thoroughly by pipetting the mixture and equilibrated for at least 5 mins with the fluorescence spectrum being measure from 350-600 nm. All the experiment were performed in technical triplicate and averaged, and performed three times as independent experiments (**N=3**). Blank subtraction was performed after each titration using the baseline subtraction tool available on CaryEclipse software. Calculations, including averaging of the triplicates, extrapolation of the maxima and plotting of the curves, were carried out in GraphPad Prism software. For the calculation of the Kd (specifically for **ODN-2, ODN-6, ODN-8, ODN-9, ODN-10, ODN-11, ODN-12** and **ODN-13**), the extrapolated fluorescence intensity value at λ=435 nm was plotted in function of the equivalents of RHAU23 added to the mixture. The values were normalised, and fitted using a Hill's specific binding curve using the integrated function of Graphpad Prism 10, obtaining the Kd values for each titration. To assess the statistical significance in case of two non-overlapping binding curves (**ODN-9** and **ODN-13**), a t-test was additionally performed. The kd values are reported as a mean of 3 independent experiments (N=3), with standard deviation (±SD), and summarised in the table below (Table S3)

*Table S3. Kd values of RHAU23 binding to the G4 DNAs using 5FU modified ODN synthesised in this study.*

| ODN | Sequence (5'→3') | Kd | Notes |
|---|---|---|---|
| **ODN-2** | T(**5FU)**GGGTGGGTGGGTGGGT | 0.33 ± 0.02 μM | 5' tetrad |
| **ODN-6** | TTGGGTGGGTGGGTGGG**(5FU)** | 1.5 ± 0.1 μM | 3' tetrad |
| **ODN-8** | T(**5FU)**GGGTGGGTGGGTGGG**(5FU)**T | 0.33 ± 0.01 μM | 5' tetrad[a] |
| | | 1.40 ± 0.1 μM | 3' tetrad[a] |
| **ODN-9** | **(5FU)**GGGCGGGCGCTAGGGAGGGT | 1.7 ± 0.1 μM | 5' tetrad |
| **ODN-10** | TGGGCGGGCGCTAGGGAGGG**(5FU)**T | 0.8 ± 0.1 μM | 3' tetrad |
| **ODN-11** | **(5FU)**GGGCGGGCGCTAGGGAGGG**(5FU)**T | 1.3 ± 0.1 μM | Not possible to discriminate the two binding events |
| **ODN-12** | AAGGGAAGGG(**5FU**)A | 0.61± 0.03 μM | Binding event 1[b] (unknown tetrad) Bimolecular G4 |
| | | 1.4 ± 0.03 μM | Binding event 2[b] (unknown tetrad) Bimolecular G4 |
| **ODN-13** | (**5FU**)GGGGT | 0.75 ± 0.07 μM | Tetramolecular G4, not possible to discriminate multiple binding events |

[a] the difference of Kd between the two distinct tetrad using a single, dually modified olignonucleotide ODN-8 is statistically significant ($p<0.001$ using paired two-tailed t-test, ***)

[b] the difference of Kd between the two distinct tetrad using a single, dually modified olignonucleotide ODN-8 is statistically significant ($p<0.001$ using paired two-tailed t-test, ***)

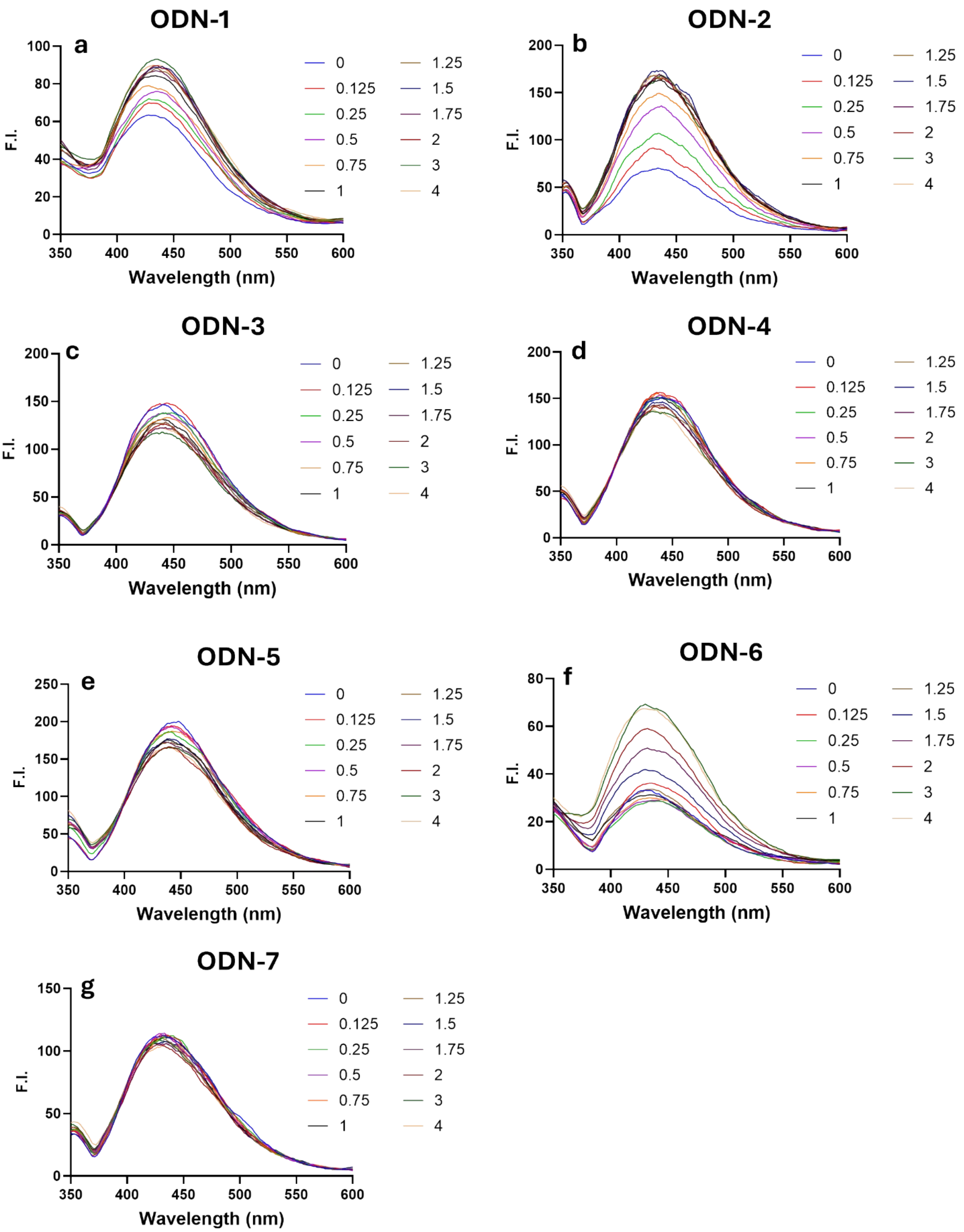

ODN-1
a
ODN-2
b
ODN-3
c
ODN-4
d
ODN-5
e
ODN-6
f
ODN-7
g
F.I.
Wavelength (nm)
0
0.125
0.25
0.5
0.75
1
1.25
1.5
1.75
2
3
4

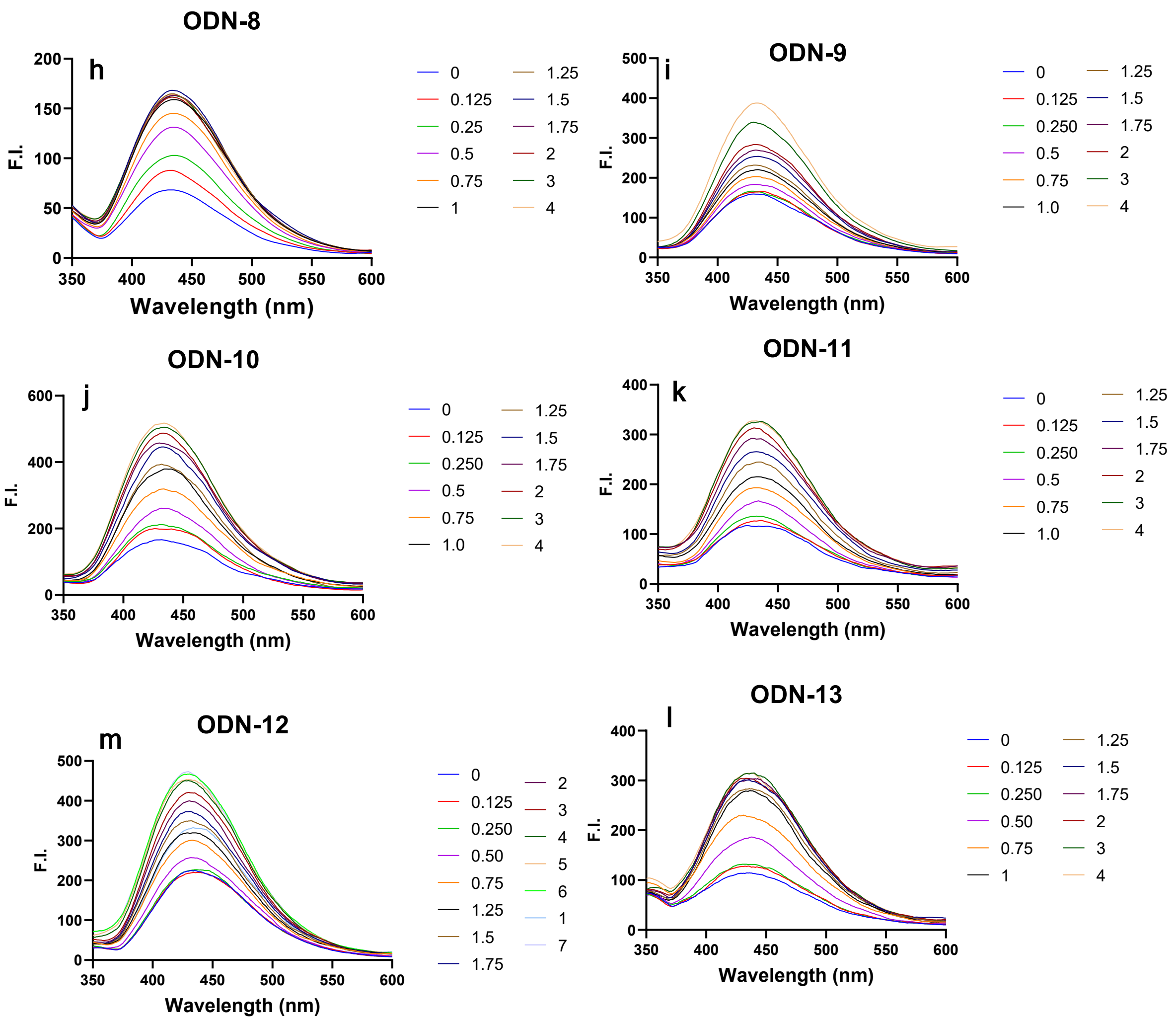


**Figure S 18**. *Titrations of Fluorescent ODNs with RHAU23, at increasing equivalents (0 to 4, with the exception of ODN-13 that was titrated to 7 eq), a.* ***ODN-1****, b.* ***ODN-2****, c.* ***ODN-3****, d.* ***ODN-4****, e.* ***ODN-5****, f.* ***ODN-6****, g.* ***ODN-7****, h.* ***ODN-8****, i.* ***ODN-9****, j.* ***ODN-10****, k.* ***ODN-11****, l.* ***ODN-12****; m.* ***ODN-13****. Experiments performed in Phosphate buffer, pH 7 (10 mM) supplemented with 70 mM KCl.*

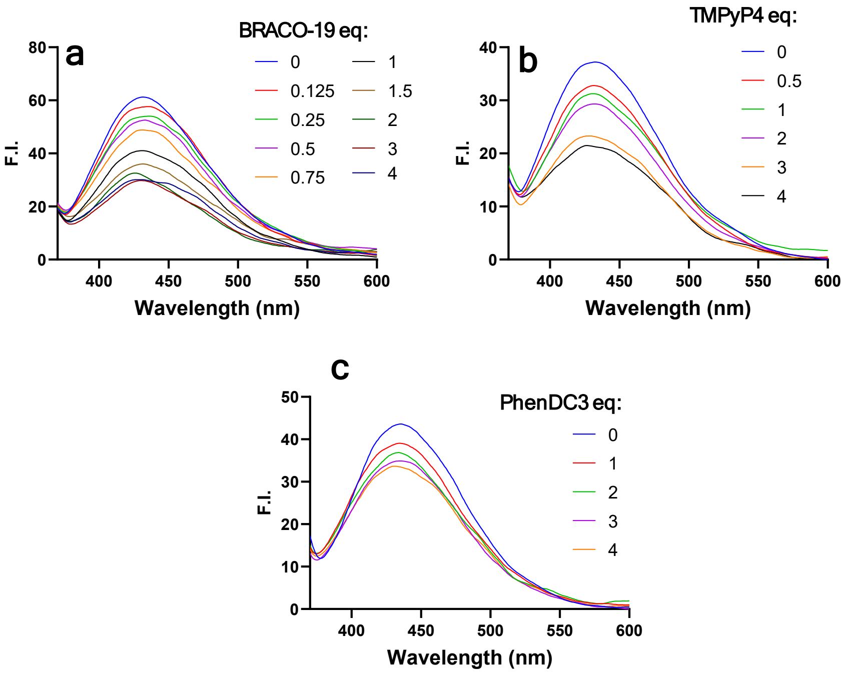


Figure S 19. *Titrations of small molecule binders with* ***ODN-2****, a. BRACO-19, b.TMPyP4, c. PhenDC3. Experiments performed in Phosphate buffer, pH 7 (10 mM) supplemented with 70 mM KCl.*

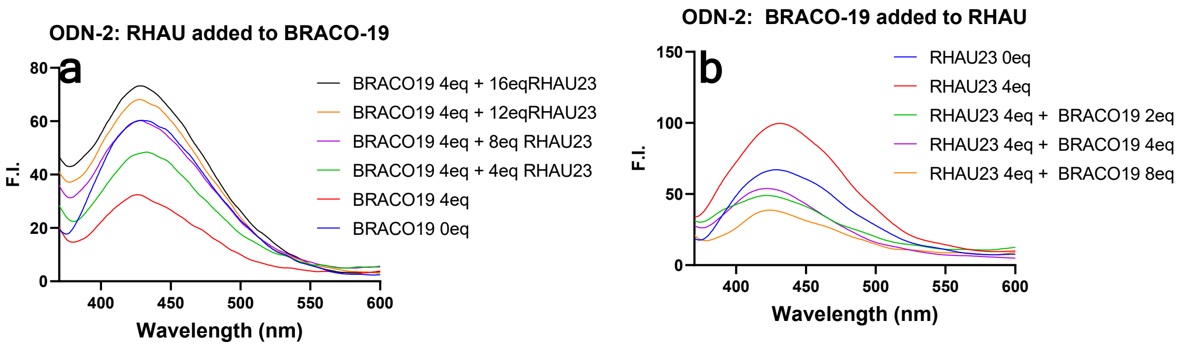


Figure S 20. *Ligand displacement titrations* ***ODN-2****, a. BRACO-19 followed by RHAU 23, b. RHAU 23 followed by BRACO-19. Experiments performed in Phosphate buffer, pH 7 (10 mM) supplemented with 70 mM KCl.*

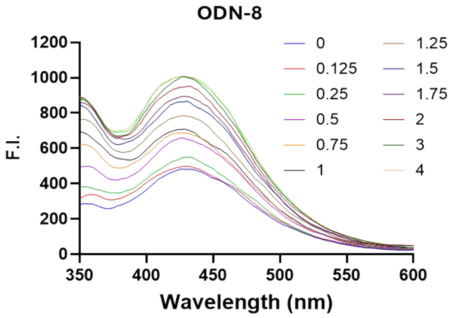


Figure S 21. *Titrations of* ***ODN-8*** *with RHAU23 under molecular crowding conditions (Identical to standard method above, i.e. Phosphate buffer, pH 7 (10 mM) supplemented with 70 mM KCl, except for the addition of PEG 600, 20 %).*

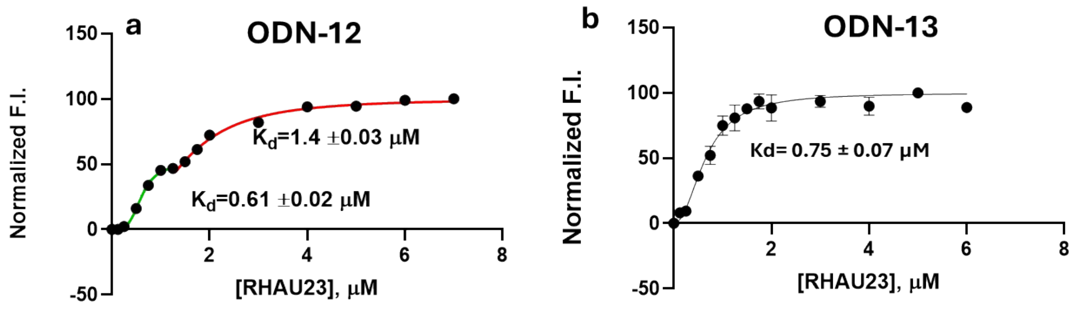


*Figure S 22. Binding curves obtained when plotting the extracted fluorescence intensity value at λ=435nm for* ***ODN-12*** *(a) and* ***ODN-13****. The values of Kd are reported in table S3.*

## 5. CD Titration Experiment

Prior to titration ODNs were annealed at 2 μM with a salt complement of KCl (70 mM) and $K_2HPO_4$ buffer (pH 7, 10 mM). 1 mL of solution was then transferred to a quartz cuvette (1 cm pathlength). The CD spectrum of the DNA was recorded in absence of any ligand, and subsequently increasing amounts of peptide were added. DNA was titrated into the solution and mixed thoroughly by pipetting the mixture and equilibrated for at least 5 mins prior recording each spectra. The experiment was performed in technical triplicate and averaged, and performed three times as independent experiments (**N=3**). Blank subtraction was performed after each titration using the baseline subtraction tool available on GraphPad prism software. Calculations, including averaging of the triplicates, extrapolation of the maxima and plotting of the curves, were carried out in GraphPad Prism software. For the calculation of the Kd, the extrapolated CD (mdeg) value at λ=264 nm (maximum of parallel G4 structure) was plotted in function of the equivalents of RHAU23 added to the mixture. The values obtained were normalised, and converted through linear rescaling using the following conversion formula:

$$\%Bound_{Xeq} = 100 - CD\ 264nm_{X\ eq}$$

Where $\%Bound_{Xeq}$ is the percentage of peptide bound to the DNA upon the addition of X equivalents, and $CD\ 264nm_{X\ eq}$ is the normalized CD value (mdeg) of the maximum at 264 nm upon addition of X equivalents of peptide.

The values obtained were fitted using a Hill's specific binding curve using the integrated function of Graphpad Prism 10, obtaining the Kd values for each titration. To assess the statistical significance of the two non-overlapping binding events, a t-test was additionally performed ($p<0.05$). The kd values are reported as a mean of 3 independent experiments (N=3), with standard deviation (±SD).

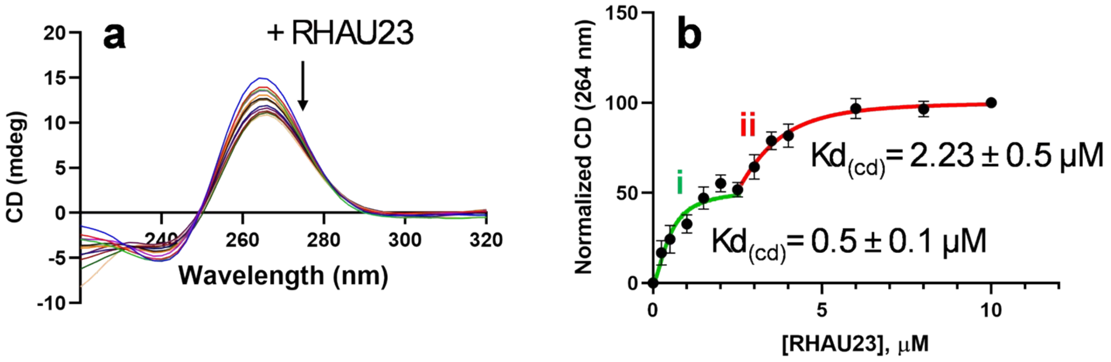


*Figure S 23. CD titration experiment performed with ODN-8 (dual modified 5FU containing oligonucleotide), titrated with increasing amounts of RHAU23 peptide. The difference among the two Kd (event i and event ii) is found statistically significant (two values were compared using a paired two-tailed t-test, p<0.05,*).*

## 6. Structures for rationalisation

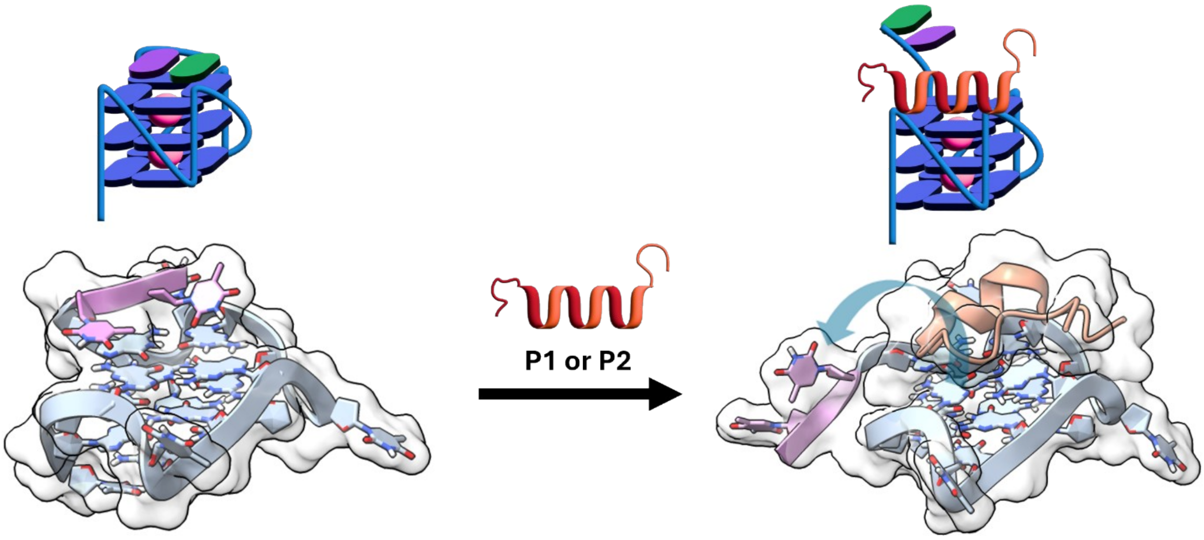


Figure S 24. Schematic representing the binding of RHAU 23 to the T95-2T G-quadruplex with the displacement of the T-caps highlighted (Adapted from PDB: 2lk7 left and PDB: 2n21 right).[3,4]

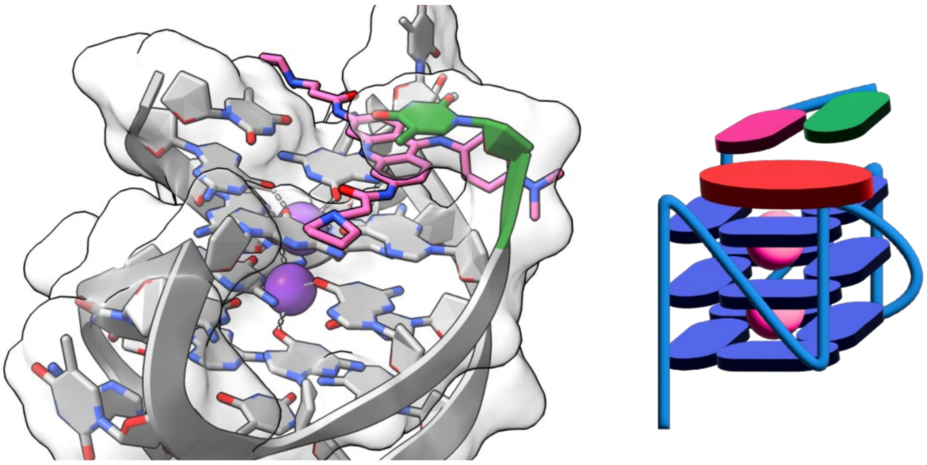

Figure S 25. An example of the binding of BRACO-19 (pink) to a telomeric sequence G-quadruplex with the stacking of the T-caps highlighted (green) (Adapted from PDB: 3ce5).[5]

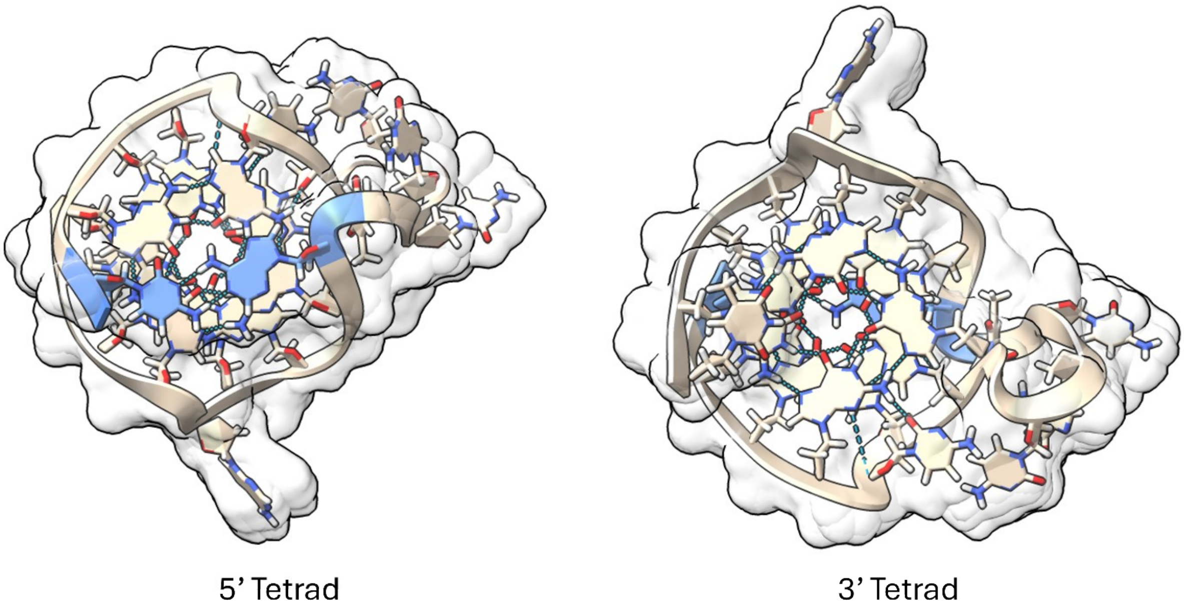


Figure S 26.: Schematic of the 5’ and 3’ tetrad of C-KIT2 G-quadruplex with blocking nucleotides highlighted (blue) (Adapted from PDB: 2kyp).[6]

## 7. References


1 A. V. Tataurov, Y. You and R. Owczarzy, Predicting ultraviolet spectrum of single stranded and double stranded deoxyribonucleic acids, *Biophys Chem*, 2008, 133, 66–70.

2 N. J. Greco and Y. Tor, Simple Fluorescent Pyrimidine Analogues Detect the Presence of DNA Abasic Sites, *J Am Chem Soc*, 2005, 127, 10784–10785.

3 B. Heddi, V. V. Cheong, H. Martadinata and A. T. Phan, Insights into G-quadruplex specific recognition by the DEAH-box helicase RHAU: Solution structure of a peptide–quadruplex complex, *Proceedings of the National Academy of Sciences*, 2015, 112, 9608–9613.

4 N. Q. Do and A. T. Phan, Monomer–Dimer Equilibrium for the 5'–5' Stacking of Propeller-Type Parallel-Stranded G-Quadruplexes: NMR Structural Study, *Chemistry – A European Journal*, 2012, 18, 14752–14759.

5 N. H. Campbell, G. N. Parkinson, A. P. Reszka and S. Neidle, Structural Basis of DNA Quadruplex Recognition by an Acridine Drug, *J Am Chem Soc*, 2008, 130, 6722–6724.

6 V. Kuryavyi, A. T. Phan and D. J. Patel, Solution structures of all parallel-stranded monomeric and dimeric G-quadruplex scaffolds of the human c-kit2 promoter, *Nucleic Acids Res*, 2010, 38, 6757–6773.
